# Biome evolution in subfamily Cercidoideae (Leguminosae): a tropical arborescent clade with a relictual depauperate temperate lineage

**DOI:** 10.1007/s40415-024-01058-z

**Published:** 2024-12-18

**Authors:** Charlotte Hagelstam-Renshaw, Jens J. Ringelberg, Carole Sinou, Warren Cardinal-McTeague, Anne Bruneau

**Affiliations:** 1 Institut de Recherche en Biologie Végétale and Département de Sciences Biologiques, Université de Montréal, Montréal, QC H1X 2B2 Canada; 2 School of Geosciences, Old College, University of Edinburgh, South Bridge, Edinburgh, EH8 9YL UK; 3 Department of Forest and Conservation Sciences, Faculty of Forestry, University of British Columbia, 2424 Main Mall, Vancouver, BC V6T 1Z4 Canada

**Keywords:** Ancestral character reconstruction, BEAST, BioGeoBEARS, Biogeography, Fabaceae, Growth form evolution, make.simmap, Phylogenetic biome conservatism

## Abstract

Some plant lineages remain within the same biome over time (biome conservatism), whereas others seem to adapt more easily to new biomes. The c. 398 species (14 genera) of subfamily Cercidoideae (Leguminosae or Fabaceae) are found in many biomes around the world, particularly in the tropical regions of South America, Asia and Africa, and display a variety of growth forms (small trees, shrubs, lianas and herbaceous perennials). Species distribution maps derived from cleaned occurrence records were compiled and compared with existing biome maps and with the literature to assign species to biomes. Rainforest (144 species), succulent (44 species), savanna (36 species), and temperate (10 species) biomes were found to be important in describing the global distribution of Cercidoideae, with many species occurring in more than one biome. Two phylogenetically isolated species-poor temperate (*Cercis*) and succulent (*Adenolobus*) biome lineages are sister to two broadly distributed species-rich tropical clades. Ancestral state reconstructions on a time-calibrated phylogeny suggest biome shifts occurred throughout the evolutionary history of the subfamily, with shifts between the succulent and rainforest biomes, from the rainforest to savanna, from the succulent to savanna biome, and one early occurring shift into (or from) the temperate biome. Of the 26 inferred shifts in biome, three are closely associated with a shift from the ancestral tree/shrub growth form to a liana or herbaceous perennial habit. Only three of the 13 inferred transcontinental dispersal events are associated with biome shifts. Overall, we find that biome shifts tend to occur within the same continent and that dispersals to new continents tend to occur within the same biome, but that nonetheless the biome-conserved and biogeographically structured Cercidoideae have been able to adapt to different environments through time.

**Supplementary Information:**

The online version contains supplementary material available at 10.1007/s40415-024-01058-z.

## Introduction

Phylogenetic studies have shown both that more closely related species tend to be more ecologically similar (Burns and Strauss 2011) and that there is a tendency for plant lineages to remain within the same biome over time (biome conservatism) (Wiens and Donoghue 2004; Wiens and Graham 2005; Crisp et al. 2009; Pennington et al. 2009; Hughes et al. 2013; Ringelberg et al. 2020; Segovia et al. 2020). Phylogenetic biome or niche conservatism can be defined as “the extent to which ​​species retain ancestral ecological traits and environmental distributions” (Crisp et al. 2009). Biome or niche conservatism has been documented, for example, in many Southern Hemisphere clades within the eucalypts, grasses and legumes (Crisp et al. 2009), in succulent biome clades (Lavin et al. 2004; Pennington et al. 2009; Gagnon et al. 2019; Ringelberg et al. 2020), in the Mediterranean climate *Medicago* (Leguminosae) (Yang et al. 2022) and in more endemic clades such as the Brazilian genus *Cryptanthus* Otto & A. Dietr. (Bromeliaceae) (Cruz et al. 2017). However, there is also evidence from other studies that in some plant lineages frequent biome shifts have occurred over time (Simon et al. 2009; Edwards and Donoghue 2013; Weeks et al. 2014; Ogburn and Edwards 2015; Cardillo et al. 2017; Schley et al. 2022), as has been suggested in the legume family (Schrire et al. 2005). Widely distributed pantropical clades make ideal candidates for studying the prevalence of biome conservatism vs. shifts as they provide a framework for understanding the dynamics of biome shifts across multiple different biomes, while at the same time taking into account the importance of oceanic dispersals. The species-rich and morphologically diverse family Leguminosae (Fabaceae) includes several pantropical clades in which phylogenetic relationships are now better resolved. Most recently, biome evolution has been studied in the Caesalpinieae (Gagnon et al. 2019) and Mimoseae (Ringelberg et al. 2023) (subfamily Caesalpinioideae), Detarioideae (Estrella et al. 2017), the Pterocarpus clade (Schley et al. 2022) and Millettioid/Phaseoloid clade (Oyebanji et al. 2023) (subfamily Papilionoideae), five pantropical clades which include species that occur in both dry and wet biomes but in which biome shifts vary in frequency and direction.

Four major biomes were described by Schrire et al. (2005) as being important in studying global distribution patterns in legumes: a temperate biome, which includes warm and cold regions of the northern and southern hemisphere, and three tropical biomes that include a rainforest biome, a grass biome and a succulent biome. The rainforest biome is characterized by a warm and humid climate with heavy rainfall (year-round precipitation/no significant dry season). The grass biome is a seasonally dry, grass-rich and fire-prone biome with a distribution range that includes savannas and occurs on all tropical continents. Here we focus on tropical grassy areas and use savanna to designate this biome. The succulent biome is also seasonally dry, but grass-poor and rich in succulent species that have specialized water-storing tissues (Oliveira-Filho et al. 2013; Ringelberg et al. 2020), does not experience regular fires, and is absent from Asia and Australia/Oceania. This biome, which includes the seasonally dry tropical forest or SDTF (Pennington et al. 2009; Särkinen et al. 2011; Cardoso et al. 2021; Fernandes et al. 2022), is a conservation priority due to high species endemism and large threatened areas (DRYFLOR et al. 2016). There are many more ways to define or delineate biomes (e.g., Higgins et al. 2016; Moncrieff et al. 2016; Mucina 2019; Ringelberg et al. 2020), including definitions based solely on climate (Mucina 2019), however, these four biomes are most relevant to the evolution of legumes and have been considered in several biogeographical studies of other legume clades (Lavin et al. 2004; Estrella et al. 2017; Gagnon et al. 2019; Ringelberg et al. 2020, 2023; Schley et al. 2022).

Many studies focusing on plant evolution have shown tendencies in the types or directions of observed biome shifts. Frequent shifts have been identified between rainforest and dry open forest biomes in some plant lineages, in particular, during the Miocene in the Neotropics where changes in climate resulted in the expansion of rainforests and contraction of dry forests, and since the Miocene in Africa where increased aridity led to the expansion of grasslands (Hughes et al. 2013; Estrella et al. 2017; Antonelli et al. 2018; Couvreur et al. 2020; Gorel et al. 2022; Schley et al. 2022). Different factors have been suggested to influence the likelihood of plants to shift biomes, including the age of biomes and exposure of lineages to new environments over time, meaning that shifts may occur more frequently between adjacent and/or more interconnected biomes, and certain morphological traits may also be pre-existent in species and facilitate transitions to new biomes (Edwards and Donoghue 2013; Donoghue and Edwards 2014; Echeverria-Londoño et al. 2018). However, to better understand what drives lineages to conserve or switch from their ancestral biomes, and better explain species distribution patterns across continents and the importance of dispersal limitation, phylogenetic biome conservatism and evolution need to be further investigated in other widespread pantropical clades.

Variation in plant life history, such as in habit or growth form, may be a trait enabling or facilitating adaptation to diverse environmental conditions (Rowe and Speck 2005). For example, Couvreur et al. (2015) suggested that shifts from a non-climbing habit to a climbing habit have played an important role in the diversification of palms in tropical rainforests. In the Caesalpinieae legumes, Gagnon et al. (2019) showed that biome shifts were infrequent but closely associated with shifts in growth form. The climbing habit in plants has evolved independently numerous times throughout angiosperms and is found in over 130 families (Gentry 1992), suggesting that lianescence is an ecologically important trait. Additionally, while many lianas are considered to be light-demanding, rapid-growing under high-light conditions and exhibit higher plasticity than less light-demanding species (Yuan et al. 2016), shade-tolerant lianescent species exist as well. This phenotypic flexibility and rapid growth could make lianas more competitive in the dense tropical forest and other variable environments in which they are often found (Yuan et al. 2016), making the lianescent habit advantageous in adapting to new biomes.

The legume subfamily Cercidoideae (Leguminosae/Fabaceae) includes 14 genera (Sinou et al. 2020) and approximately 398 species (LPWG 2021), distributed across all four biomes described in Schrire et al. (2005), with a large number of species found in the tropical regions of South America, Asia and Africa (LPWG 2017). Species can have an arborescent, shrubby, lianescent or herbaceous (i.e., prostrate herbaceous perennials, only in *Tylosema* (Schweinf.) Torre & Hillc.) habit, mostly unarmed but sometimes with tendrils or spines. In a study focused on three Asian liana species of *Phanera* Lour. and *Cheniella* R.Clark & Mackinder and two Asian tree species of *Bauhinia* L. in Cercidoideae, Cai et al. (2007) reported that the light-demanding lianas grow faster than the light-demanding trees and that this is the result of higher specific leaf area, leaf mass ratio and leaf area ratio in lianas. However, it remains unclear to what extent habit shifts have co-occurred with biome shifts in Cercidoideae, and whether differences in growth form can lead to phenotypic advantages that would facilitate changes in biomes. As Cercidoideae species occur in a variety of biomes on almost all continents and have distinct habits, this is an ideal group for investigating the prevalence of biome conservatism vs. shifts and the direction of shifts. The phylogenetic analyses of Sinou et al. (2020) based on plastid and duplicated copies of the nuclear *Legcyc* locus suggested that multiple biome shifts and continental disjunctions have occurred in Cercidoideae, but these patterns have not been fully examined and further investigation is necessary to better understand whether and how shifts in biome and biogeography are related.

The main objective of this study is to examine biome shifts in a phylogenetic context, determining the extent to which there have been biome shifts or biome conservatism throughout the evolution of Cercidoideae lineages and identifying pairs of biomes between which we deduce more frequent shifts than others. We also consider the direction of biome shifts as well as their associations with patterns of habit evolution and biogeographical history. We test the hypotheses that (i) Cercidoideae lineages exhibit multiple biome shifts throughout their evolution, (ii) that these shifts occur most often between the succulent and savanna biomes and between the rainforest and savanna biomes, which are the most geographically close and are often interconnected, and (iii) that lianescent lineages have shifted biomes more frequently than non-lianescent lineages, given the potential advantage of their phenotypic flexibility and rapid growth in new or variable environments. Studying biome evolution concomitantly with habit evolution and historical biogeography can lead to better understanding of the relative importance of phylogenetic biome conservatism versus dispersal limitation in explaining the distribution and biodiversity of globally distributed plant clades. Understanding these phenomena can help explain not only the global distribution patterns that we observe in extant species but understanding if species can switch biomes or not can also provide insight into the survival and potential invasiveness or vulnerability of species under climate change.

## Materials and methods

### Species occurrence data

– The names and taxonomy of all species of Cercidoideae were verified as described in le Roux et al. (2022) and integrated in the LPWG (2021) checklist (available on the Catalogue of Life ChecklistBank (https://data.catalogueoflife.org/dataset/2304/about) and on the Legume Data Portal (https://www.legumedata.org/taxonomy/browse)). Occurrence records were downloaded from the *Global Biodiversity Information Facility* (GBIF 2020) (https://www.gbif.org/), *Latin American Seasonally Dry Tropical Forest Floristic Network* (DryFlor, http://www.dryflor.info/), *Southwestern Environmental Information Network* (SEINet, https://swbiodiversity.org/) and *speciesLink* (http://www.splink.org.br/) for 14 genera in Cercidoideae: *Adenolobus* (Harv. ex Benth.) Torre & Hillc. (GBIF 10.15468/dl.3fv3ph), *Barklya* F.Muell. (GBIF 10.15468/dl.s483xj), *Bauhinia* L. (GBIF 10.15468/dl.epcb8p), *Brenierea* Humbert (GBIF 10.15468/dl.tvjv2m), *Cercis* L. (GBIF 10.15468/dl.6xnttt), *Cheniella* R.Clark & Mackinder (GBIF 10.15468/dl.z229hg), *Gigasiphon* Drake (GBIF 10.15468/dl.e2nfd9), *Griffonia* Baill. (GBIF 10.15468/dl.k4enf3), *Lysiphyllum* (Benth.) de Wit (GBIF 10.15468/dl.22qy3f), *Phanera* Lour. (GBIF 10.15468/dl.as44t9), *Piliostigma* Hochst. (GBIF 10.15468/dl.3s2hq5), *Schnella* Raddi (GBIF 10.15468/dl.mxdh5m), *Tournaya* A.Schmitz (GBIF 10.15468/dl.mezxk8) and *Tylosema* (Schweinf.) Torre & Hillc. (GBIF 10.15468/dl.teh76e). Records from different sources were merged to have a single dataset per genus. These records were then subjected to an extensive data cleaning process in order to match each occurrence to their accepted name according to our species list as well as to remove doubtful occurrences. This included removing occurrence records that were not associated with preserved specimens (mostly observations), that had missing or imprecise coordinates (degree-level only or with a latitude or longitude of exactly zero) or coordinates that are those of country (or regional) centroids or botanical gardens. Records identified as being cultivated or introduced were also removed, and the “notes” and “locality” columns that exist in the datasets were also searched for keywords such as “botanical garden”, “garden”, “ornamental” or “cultivated”. Species-level maps were produced to check individual occurrence records for outliers such as occurrences located in oceans (using the identify function in R to check individual occurrences). The occurrence datasets were cleaned using R (R Core Team 2021) and the cleaning process was based on R scripts from Gagnon et al. (2019) and Ringelberg et al. (2020).

After removing duplicate records (i.e. occurrences with the same coordinates), we obtained a total of 26,946 occurrence records for 339 species and subspecies in Cercidoideae. Occurrence maps were produced in R for each species using the ggplot2 (Wickham 2016) and maps (Becker and Wilks 2021) R packages.

### Biome maps

– Occurrence maps were compared with existing biome maps to determine to which biome each occurrence point (geographic coordinates) belongs and this information was considered when attributing individual species to biomes (see next section). Three georeferenced biome maps were considered: a map of the tropical rainforest biome (Corlett and Primack 2011), a map of the succulent biome (Ringelberg et al. 2020), and a map of the grass biome (Lehmann et al. 2019). Lehmann et al.’s map of the globally distributed grass biome includes the tropical savanna biome as well as temperate grasslands (e.g., of North America and Northern China), but following Schire et al. (2005), we do not include the temperate grasslands in our definition of the savanna biome. We removed these temperate grasslands from the Lehmann et al. grass map following criterion 4 (“all areas where temperatures do not go below freezing in a typical year”) of Feeley and Stroud (2018) for the distribution of the tropics. Therefore, the 14 species and subspecies in the genus *Cercis*, which are not tropical (Davis et al. 2002), were assigned to a general temperate biome.

As our objective was to determine the number of occurrences in each biome, we had to modify these three maps for our purposes. The rainforest and savanna maps are binary, whereas the succulent biome map is continuous, with each grid cell representing the fraction of models that predict the occurrence of stem succulents (see Ringelberg et al. 2020). To match the other biome maps, we converted the succulent biome map to binary using a threshold of 33%, which was the threshold used by Ringelberg et al. (2020) to convert their map to binary. In addition, because the succulent biome map is at a coarser resolution (0.25 deg) than the rainforest and grass maps (0.1 deg), the latter two were converted to the 0.25 deg resolution using the disaggregate function in the raster R package (Hijmans and van Etten 2012).

All three biome maps had some areas of overlap with each other. The areas of overlap between the rainforest and succulent biome maps were smaller and more localized, whereas the grass biome map overlapped greatly with both of these regions. Three significant areas of overlap between the rainforest and succulent biome maps were identified: an area along the Atlantic coast of Brazil most likely belonging to the rainforest biome (Roesch et al. 2009); a small region in south-eastern Madagascar belonging to the rainforest biome (Du Puy and Moat 2002); and a small region close to the southern border of Mexico most likely corresponding to the rainforest biome (Urbazaev et al. 2018). Additionally, Cercidoideae species that occur within these small areas of overlap were most often reported as rainforest species, according to literature. We therefore removed these areas of overlap from a modified succulent biome map and considered them to belong to the rainforest biome. Areas of overlap between the grass and succulent biome maps were particularly large. Following Ringelberg et al. (2020), who more precisely modeled the global distribution of the succulent biome based on the presence of stem succulents, we removed these areas of overlap from the savanna biome map. Large areas of overlap also exist between the rainforest and grass biome maps, particularly in the Guineo-Congolian region as well in the Amazonian Rainforest. It was more challenging to determine whether these areas should be described as part of the rainforest or savanna biomes, in part due the effects of recent anthropic factors such as deforestation and because these could be transition zones or “savanna-rainforest mosaics” (Aleman et al. 2020). Aleman et al. (2020) also indicated that coexistence of rainforest and savanna in tropical Africa is limited and that African forests and savannas have distinct floristic composition. Many of these areas of overlap, including those belonging to the Guineo-Congolian region and the Amazonian Rainforest, have been subject to recent anthropic pressures, and the resulting disturbed environment tends to be a drier environment resembling the tropical savanna (Aleman et al. 2020). For this reason, we considered these areas of overlap as part of the rainforest biome and removed them from the Lehmann et al. map. We therefore used three maps to represent the tropical biomes: the succulent biome map (Ringelberg et al. 2020) without areas of overlap with the rainforest biome map (Corlett and Primack 2011), the grass biome map (Lehmann et al. 2019) without areas of overlap with the succulent and rainforest maps and areas outside the tropics, and the rainforest map from Corlett and Primack (2011) without modifications. We did not use maps to assign species to the temperate biome because we could clearly establish based on literature information and occurrence data that species of *Cercis* occur in extra-tropical regions as defined by Feeley and Stroud (2018).

### Attributing species to biomes

– Using the species occurrence datasets and the three biome maps, we calculated the number of occurrences for 339 species of Cercidoideae found within four areas: only succulent biome, only rainforest biome, only savanna biome and outside of these three biomes (i.e., extra-tropical regions). Species-level maps of occurrences were produced for each biome and the proportions of occurrences in each were calculated. Finally, we produced generic-level maps of occurrences across these biomes, indicating the distribution of individual species, except for the large genera *Bauhinia*, *Schnella* and *Phanera* for which maps are shown only at the generic level.

Each species was assigned to one or several biomes by taking into account species occurrences on the biome maps and information found on herbarium specimen labels from digitized images, in floras, monographs and other literature using terms frequently encountered in literature for each of these biomes (Hagelstam Renshaw 2022). We also calculated the proportions of occurrences for each species in the four biomes. Following Dale et al. (2021), we considered species to be potentially assigned to one or several biomes based on a low threshold (i.e., when more than 10% of occurrences for a species were found within the limits of the map of the biome).

There is support for allowing species to be assigned to multiple biomes in analyses of biome conservatism and biome shifts (Dale et al. 2021). Although most species were assigned to one or two biomes, a few species were assigned to three biomes. For species with more than 10% of occurrences falling within the rainforest-succulent overlap, we compared the species occurrence maps with other more detailed regional vegetation maps and considered these additional sources of information to attribute these species to biomes. It is worth noting that a species which occurs in more than one biome should, in theory, have occurrences within the areas of overlap, as well as in the individual biomes. We took this into account when attributing these species to biomes by looking at their overall distribution and the proportions of occurrences in the savanna, rainforest and succulent biome maps, as well as the proportions outside of these three areas. When attributing biomes based on proportions of occurrences per biome, we also considered the total number of occurrences available for each species, because we could expect attribution based on overall proportions per biome to be less reliable for species with few occurrence records.

### Determining species growth forms

– Species were assigned to one of three habit categories: tree/shrub, liana or herbaceous perennial. Habit was attributed to species based on information found on herbarium specimen labels from digitized images, in floras, monographs and other literature.

### Assigning species to continents

– Species were assigned to one or several of eight continental regions based on our occurrence maps and information from literature: Africa, Madagascar, Australia, Asia, Northern Asia, North America, Central America, and South America. Species were assigned to Northern Asia when occurrences were found only within China (i.e., temperate species of *Cercis*), as occurrences for all other Asian species in Cercidoideae were either more widespread across Asia or restricted to more southern and tropical regions.

**Fig. 1:**
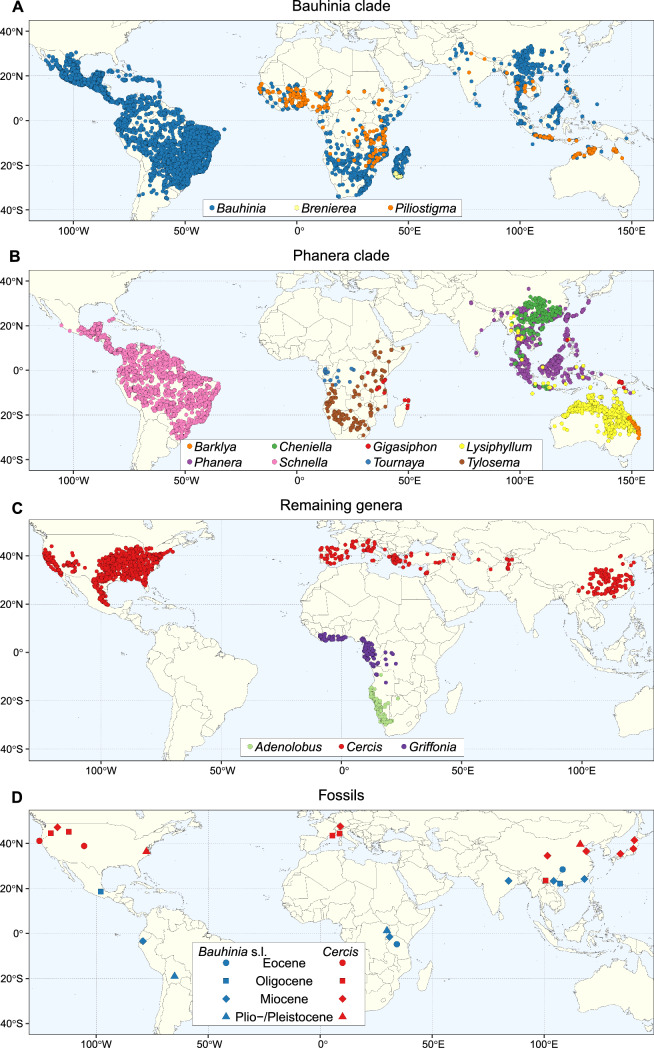
Distribution maps of Cercidoideae based on quality controlled and cleaned specimen occurrence records. A. Bauhinia clade species. B. Phanera clade species. C. Other genera. D. Distribution of *Cercis* and *Bauhinia* s.l. fossils as described in Heer (1855), Saporta (1862, 1873), Berry (1917, 1926), Principi (1916), Knowlton (1926), Chaney and Daugherty (1933), Brown (1937), Hu and Chaney (1940), Suzuki (1958), Tanai (1961), Tanai and Suzuki (1963, 1972), Becker (1972), Van Neer (1983), Bande and Srivastava (1990), Guo and Zhou (1992), Eisenmann (1994), Meyer and Manchester (1997), Kowalski (2001), Calvillo-Canadell and Cevallos-Ferriz (2002), Jacobs and Herendeen (2004), Chen and Zhang (2005), Jia and Manchester (2014), Meng et al. (2014), Wang et al. (2014), Jacques et al. (2015), Lin et al. (2015), Li et al. (2021), Jia et al. (2022), Wang et al. (2022)

We also collated from the literature information on the geographic distribution and age of fossils for Cercidoideae (summarized in Jia and Manchester 2014; Meng et al. 2014; Lin et al. 2015; Li et al. 2021; Jia et al. 2022; other references in Fig. 1 legend).

### Time-calibrated phylogeny

– To build a phylogenetic framework to evaluate biome evolution in subfamily Cercidoideae, we used the concatenated DNA sequence alignments from Sinou et al. (2020; archived on TreeBASE S25776), which included sequences for four loci (plastid *matK* and *trnL-F* and two copies of the nuclear gene *Legcyc*) and 123 accessions representing 110 species. After merging or removing multiple accessions for the same species, and removing three species because of identification or synonymy issues, the final dataset included 107 species (and 107 accessions) out of c. 398 species or 27% of Cercidoideae species, representing all genera and covering all continents and biomes in which Cercidoideae species occur. The smaller genera *Adenolobus* (2 species)*, Barklya* (1 sp.)*, Brenierea* (1 sp.) and *Tournaya* (1 sp.) have 100% species coverage. Other genera vary from 15–80% species coverage: *Bauhinia* (55 species /c. 195 species), *Cercis* (5 spp /10 spp)*, Cheniella* (3 spp /10 spp)*, Gigasiphon* (2 spp /5 spp)*, Griffonia* (1 sp. /4 spp)*, Lysiphyllum* (6 spp /9 spp)*, Phanera* (14 spp /103 spp), *Piliostigma* (2 spp /5 spp)*, Schnella* (10 spp /47 spp), and *Tylosema* (4 spp /5 spp).

A time-calibrated phylogeny was reconstructed using BEAST v. 2.6.3 (Bouckaert et al. 2019) with four partitions. We identified the TN93 + F + G4 model based on the Akaike information criterion using Modelfinder (Kalyaanamoorthy et al. 2017) as implemented in IQ-TREE (Nguyen et al. 2015) as being the best model for *Legcyc*1, *Legcyc*2, and the TVM + F + G4 as being the best for *matK* and *trnL-F*. Because the TVM model is not available in BEAST, we used the more complex GTR model. We ran the BEAST analyses using the TN93 model for the first two partitions and the GTR model for the last two partitions and compared these results with those obtained using a GTR model for all four partitions. These changes seemed to make little difference on the age estimates, which is why we used the more complex GTR substitution model for all four partitions. We used a gamma site model. Clock models and tree models were linked for these four partitions and their site models were not linked. The analysis was run for 15 million generations and trees were sampled every 5,000 generations. Independent runs were verified as converged and well-sampled (effective sample size > 200) using Tracer v1.7.2 (Rambaut et al. 2018) and a burn-in was estimated at 0.25 (3,750,000 generations). We used two fossil priors: *Cercis* leaf and fruit fossils found in Oregon with a minimum age of 36 Ma (Herendeen et al. 1992; Jia and Manchester 2014) placed on the stem node for the genus (Davis et al. 2002; Fritsch and Cruz 2012), and a *Bauhinia* leaf fossil found in Tanzania and dated to a maximum of 46 Ma (Jacobs and Herendeen 2004) placed at the stem node of the clade including all Cercidoideae species except *Cercis* and *Adenolobus* (Bruneau et al. 2008). We applied a log normal relaxed clock and a birth–death model to these two fossil priors, and we rooted the tree with the *Cercis* clade.

### Ancestral character reconstruction and biome evolution

– Ancestral character reconstruction analyses were done for the 107 species included in the phylogeny. Ancestral biomes and habits were inferred using BEAST v. 2.6.3 (Bouckaert et al. 2019) and stochastic character mapping (Revell 2012). Because ancestral character reconstructions are very likely to be imperfect due to extinction of lineages (Marshall 2017), we chose to use more than one method of reconstruction to account for the shortcomings of these approaches and to attempt to draw general conclusions from their outcomes.

To accommodate species that had been attributed to more than one biome in BEAST, we followed Gagnon et al. (2019) and manually edited the xml file to include ambiguity codes for characters with polymorphic states. Parameters for the BEAST reconstruction analyses were set in BEAUTi. The two discrete characters, ie. biomes and habit, were added as two additional partitions sharing the same tree model with the other four partitions but not sharing the same clock and substitution (site) models. Each character had its own clock and site models, which were kept as default parameters (gamma site model and strict clock model). The BEAST analysis included four replicate MCMC chains of 15 million generations, sampling every 5,000 generations. The biome topologies and habit topologies resulting from the four replicates were combined in LogCombiner v2.6.6 (Bouckaert et al. 2019). Maximum clade credibility trees were generated in TreeAnnotator v2.6.6 (Bouckaert et al. 2019) using median node heights and viewed in FigTree v1.4.4 (Bouckaert et al. 2019).

Ancestral biomes and habits were also inferred using stochastic character mapping with the function make.simmap in the phytools R package (Revell 2012). This method is appropriate as it allows for polymorphic/missing character states. Species assigned to multiple biomes were given an equal probability of belonging to each of the biomes to which they were assigned, and these probabilities were used to simulate character mapping. Model fitting was performed using the fitMk function in phytools (Revell 2012); we considered an equal-rates model (ER), a symmetrical model (SYM) and an all-rates-different model (ARD) and chose the best-fitted model for biomes and habit using the Akaike information criterion (Akaike 1974). We then performed stochastic character mapping for biomes and habits on the maximum clade credibility tree using make.simmap with 500 simulations.

### Ancestral area reconstruction

– Ancestral area reconstruction was performed using BioGeoBEARS (Matzke 2013, 2014). All species were assigned to one (and some species to two or three) of eight areas (Africa, Madagascar, Asia, Northern Asia, Australia, North America, Central America, and South America). We set the maximum number of areas that can be occupied simultaneously to four, compared the fit of six models (DEC, DEC + j, DIVALIKE, DIVALIKE + j, BAYAREALIKE, and BAYAREALIKE + j), and extracted ancestral area reconstructions from the best fitting model.

### Biome shifts and phylogenetic biome conservatism

– The number of biome shifts was evaluated based on both the BEAST and make.simmap reconstructions. Shifts were inferred to have taken place along branches that connect two nodes with contrasting most probable reconstructed biome states. Shifts along branches leading to polymorphic tips in the phylogeny (i.e., taxa occurring in more than one biome) were only considered if the parental most probable reconstructed biome state was not among the tip biome states. For example, if a parental node reconstructed as most likely occurring in savanna gave rise to a tip (i.e., taxon) occurring in savanna and rainforest, this was considered as a range expansion rather than a biome shift, and not included in subsequent analyses.

To test for biome conservatism, we followed Gagnon et al. (2019) and compared the number of shifts per tree with the number of shifts per tree obtained with randomly reordered tip states using the sample function in R (null distribution) and repeating this analysis five times (using a sample of ten trees and ten make.simmap simulations). A lower number of shifts obtained with our data compared with the number of shifts with randomized data can indicate that the considered trait has some historical inertia (Maddison and Slatkin 1991), providing evidence for biome conservatism. This analysis was performed using the geiger (Pennell et al. 2014) and phytools R packages (Revell 2012).

Phylogenetic signal for biomes was calculated using two different measures: Pagel’s lambda (Pagel 1999) using the fitDiscrete function in the geiger R package (Pennell et al. 2014) and the Delta statistic (Borges et al. 2019). fitDiscrete was run using the ER, SYM and ARD models. Because fitDiscrete does not allow for missing or polymorphic character states, and 55 taxa were attributed to more than one biome, we repeated this analysis 100 times (on 100 trees), each time randomly assigning these 55 taxa to one of their biomes. Delta statistics were calculated using the observed biome and habit data, and compared to Delta statistics obtained by reshuffling the tip states 100 times. We obtained a p-value by counting the number of times that the Delta statistic for reshuffled data is superior to the Delta for the observed data. A p-value of less than 0.05 can provide evidence for phylogenetic signal (Pennell et al. 2014). High phylogenetic signal for biomes constitutes evidence for phylogenetic biome conservatism (Losos 2008).

### Biome shifts through time

– Using the output of the BEAST and make.simmap reconstructions, the relative occurrence of biome shifts through time was calculated in two different ways. Both methods assess relative numbers of shifts per five million year periods. The first approach calculates the numbers of shifts relative to the total amount of branch lengths per time period. To account for uncertainty about the exact timing of the shifts, we randomly placed each shift 1,000 times along its branch, and report both the mean and the standard deviation of the numbers of shifts per time period. The second approach calculates the numbers of shifts relative to the total number of speciation events (i.e., nodes in the phylogeny) per time period, following Ringelberg et al. (2023). For this approach shifts were placed halfway along each branch.

Comparing biome shifts through time between studies is challenging because of differences in methodology and sampling caveats (e.g., Dale et al. 2021). For this reason, in order to allow comparison of results found in Cercidoideae, we used data from Ringelberg et al. (2023) to also calculate shifts through time in another legume group, tribe Mimoseae (subfamily Caesalpinioideae), using the same two methods as above. Rather than biome shifts, shifts in Mimoseae represents shifts between major precipitation categories (see Ringelberg et al. (2023) for details).

## Results

### Geographic distribution of Cercidoideae across biomes

**Fig. 2:**
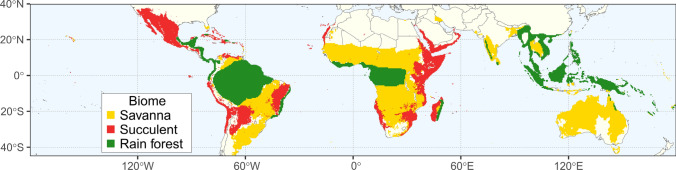
Map of biomes. Corlett and Primack’s (2011) tropical rainforest map without modifications (in green), Ringelberg et al.’s (2020) succulent biome map without areas of overlap with Corlett and Primack’s (2011) rainforest biome map (in red) and Lehmann et al.’s (2019) grass biome map without areas of overlap with the succulent and rainforest maps (in yellow, called the savanna biome here), and retaining only regions in the tropics, using criterion 4 of Feeley and Stroud (2018) for defining tropical regions (see text). Areas that are covered by none of the three biome maps correspond to other biomes, including the temperate biome

**Fig. 3:**
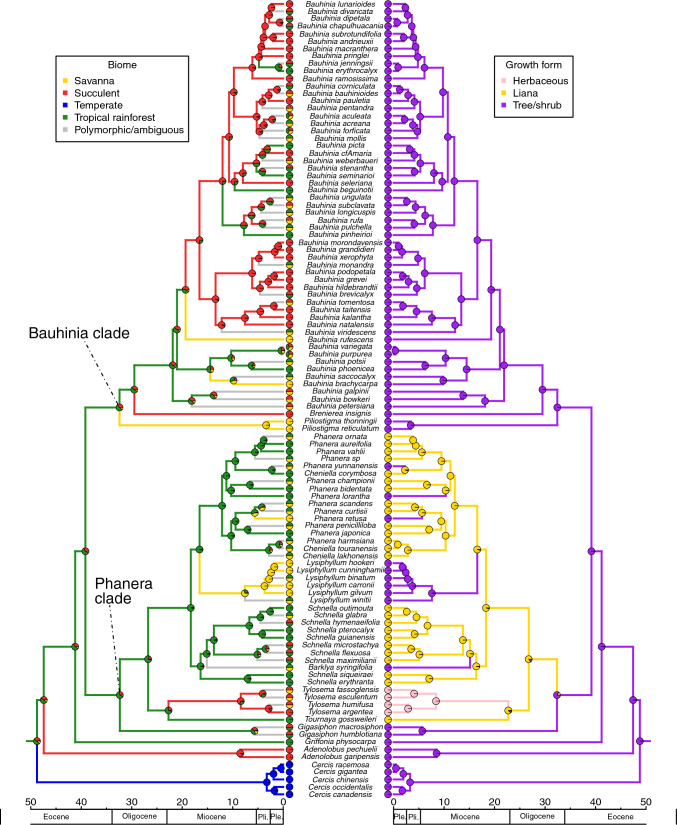
Bayesian ancestral state reconstruction for biomes (left) and habit (right) displayed on the time-calibrated phylogeny of Cercidoideae (maximum clade credibility tree), generated in BEAST v. 2.6.3. Pie charts at the tips represent the biomes and habits attributed to taxa, grey branches indicate polymorphic taxa or ambiguity in the reconstruction for the node subtended by the branch

**Fig. 4:**
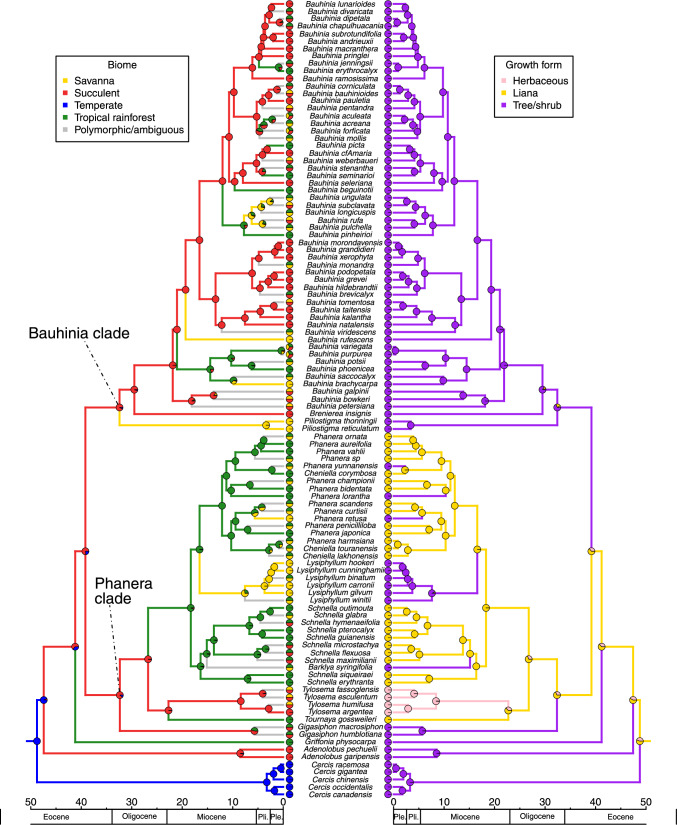
Stochastic character mapping for biomes (left) and habit (right) using make.simmap (Phytools), using the maximum clade credibility tree generated in BEAST, an all-rates-different model and 500 make.simmap simulations. Species assigned to multiple biomes were given an equal probability of belonging to each of the biomes to which they were assigned. Biome analyses have 28.798 changes between states on average and four types of shifts occurred: across 500 simulations, an average of 16.31 shifts occurred from succulent to rainforest, 8.75 from rainforest to savanna, 2.83 from temperate to succulent and 0.91 from succulent to savanna. Growth form analyses have 13.032 changes between states on average and four types of shifts occurred: across 500 simulations, an average of 8.94 shifts occurred from liana to tree/shrub, 1.64 from herbaceous to tree/shrub, 1.48 from herbaceous to liana and 0.97 from liana to herbaceous

**Fig. 5:**
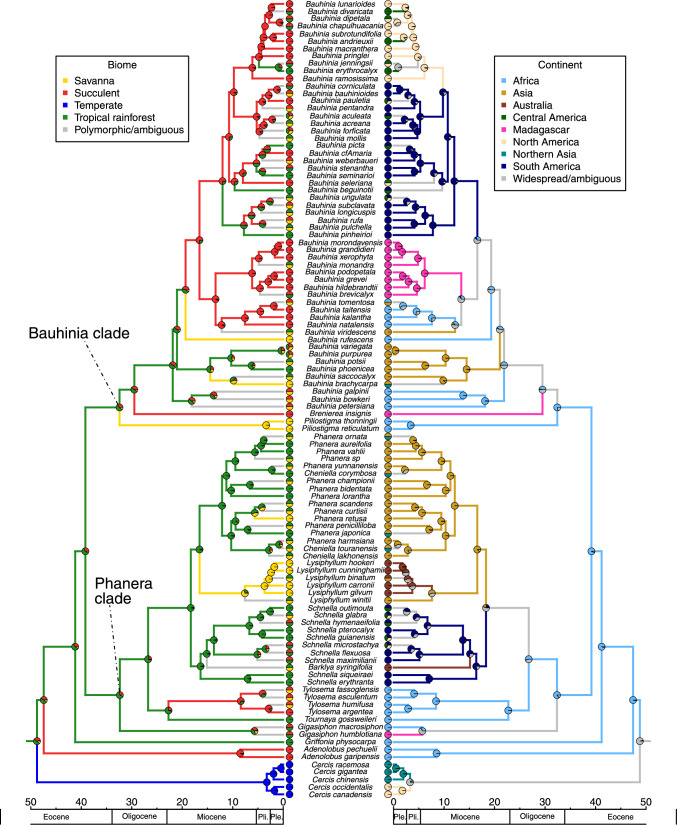
Bayesian ancestral state reconstruction for biomes (left; generated in BEAST) and biogeographic reconstruction of continents (right; generated using BioGeoBEARS) displayed on the time-calibrated phylogeny of Cercidoideae (maximum clade credibility tree). Pie charts at the tips represent the biomes and continents attributed to taxa, pie charts on nodes represent ancestral state reconstructions. All reconstructions of widespread ranges (i.e., occurring in more than one region, such as Africa and Asia) are shown in grey. Colours of branches reflect the state of the node or tip subtended by the branch (the observed state in case of tips, the reconstructed state with the highest probability in case of nodes)

– The maps (Figs. 1, 2; Supplemental Information, Figs S3–16) produced with cleaned and quality-controlled occurrence data show clearly the global distribution of Cercidoideae species in the four biomes studied. Biomes attributed to the species included in the Cercidoideae phylogeny are also shown as pie charts at branch tips (Figs. 3—5), and those for additional species not included in our phylogeny are given in Supplemental Information Figs S3-16. In the Bauhinia clade (see below; Fig. 1A), *Bauhinia* species are found primarily throughout Central America, South America and the Caribbean, many species occur in Africa south of the Sahara Desert, in Madagascar, throughout southern Asia and along the northern coast of Australia. The 166 *Bauhinia* species included (of a total of c. 195 species) are found in the rainforest (c. 64 spp), succulent (c. 65 spp) and savanna (c. 68 spp) biomes (c. 48 species are coded as occurring in more than one biome). Of the three species in *Piliostigma* for which we have occurrence records (two missing), two occur across the savanna biome of Africa and one occurs in the savanna biome in southern Asia and northern Australia. Finally, the single species of *Brenierea*, *B. insignis* Humbert, is found in the succulent biome of southern Madagascar. In the Phanera clade (Fig. 1B), the 73 *Phanera* species included (of a total of 103 spp) are found throughout rainforest (c. 63 spp) and savanna (c. 21 spp) biomes of Southeast Asia and southern China. The 47 *Schnella* species included (of a total of 47) occur from southern Mexico to southern Brazil, mostly in the rainforest, but some species also occur in the succulent and savanna biomes. The nine *Cheniella* species included (of a total of 10 spp) occur in both rainforest (4 spp) and savanna (1 sp.) biomes (4 spp in both biomes) from southern China to Indonesia. The nine *Lysiphyllum* species occur primarily in northern and central Australia, with a few species occurring in Southeast Asia, and are primarily savanna species (6–7 spp), with a few occurring in both savanna and rainforest biomes (1–2 spp). The four *Tylosema* species included (one missing) occur in the savanna (3 spp) and succulent biomes (2 spp) of southern and western Africa (1 sp. occurs in both biomes). The four *Gigasiphon* included (one missing) occur in the succulent biome of eastern Africa (1 sp.), and in the rainforest biome (3 spp), one in Madagascar, one in the Philippines and one in Papua New Guinea. The single species of *Tournaya*, *T. gossweileri* (Baker f.) A.Schmitz, is found in the rainforest biome of western Africa and the single species of *Barklya*, *B. syringifolia* F.Muell.*,* is found along the southeastern coast of Australia in the savanna biome. The remaining Cercidoideae genera (Fig. 1C) include *Cercis*, *Adenolobus* and *Griffonia*. The ten *Cercis* species occur in the temperate biome, primarily in North America (southern Canada to Mexico) and China, with two species occurring across Mediterranean Europe. The two *Adenolobus* species are restricted to the succulent biome of south-west Africa. All four *Griffonia* species occur in the rainforest biome of western Africa.

### Time-calibrated phylogeny

– Our analysis estimated the crown age of Cercidoideae in the early Eocene at 48.74 Ma (95% highest posterior density: 46.12–54.75 Ma) (Fig. 3, Supplemental Information Fig. S1). Using *Cercis* to root the phylogeny, we find strong support for two large lineages: a Bauhinia clade (Bayesian posterior probability (BPP) of 1) that includes species of *Piliostigma* sister to *Brenierea* + *Bauhinia* (BPP of 1), and a Phanera clade (BPP of 1) with the genera *Gigasiphon*, *Cheniella*, *Tournaya*, *Tylosema, Barklya, Schnella, Lysiphyllum* and *Phanera* (*Gigasiphon* as sister to the rest of the clade, BPP of 1)*. Griffonia* is resolved as sister to the clade comprising the Bauhinia and Phanera clades, and *Adenolobus* is resolved as sister to the rest of Cercidoideae excluding *Cercis* (BPP of 1). Most genera are supported as monophyletic, with two exceptions: *Cheniella* is resolved as polyphyletic (but see Gu et al. 2024) and embedded in *Phanera,* and *Schnella* is resolved as paraphyletic because of the nested position of *Barklya syringifolia*. These relationships are very similar to those found in the phylogeny of Sinou et al. (2020).

### Ancestral biomes and growth forms

**Fig. 6:**
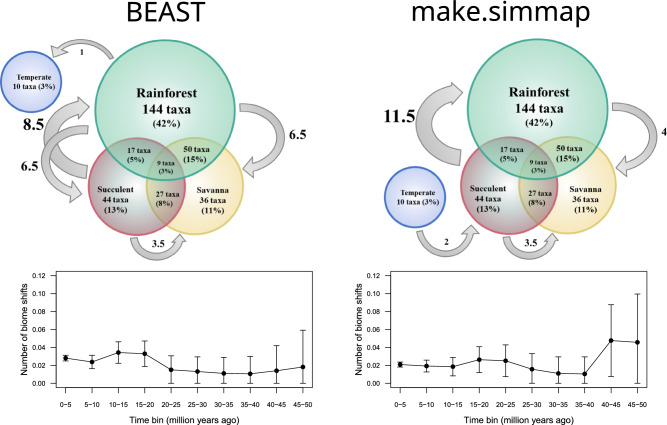
Direction and number of changes between biomes and number of biome shifts per five million years in Cercidoideae based on BEAST Bayesian ancestral character reconstruction (left) and make.simmap stochastic character mapping (right) analyses. Top: Number of Cercidoideae species assigned to each of the four biomes and percentage of taxa assigned to each biome or to multiple biomes, out of a total 337, of which 103 were assigned to more than one biome (50 taxa were assigned to both rainforest and savanna biomes, 27 taxa were assigned to both succulent and savanna biomes, 17 taxa were assigned to both rainforest and succulent biomes and nine taxa were assigned to all three of these biomes). Grey arrows represent biome shifts and direction of shifts. Shifts to more than one biome are counted half (e.g., a shift to a taxon occurring in savanna and rainforest counts as half a savanna and half a rainforest shift). Bottom: Biome shifts through time, calculated as the number of shifts divided by total branch lengths within each five million year period. To account for uncertainty about timing and phylogenetic placement of biome shifts, shifts were placed randomly along branches 1,000 times. Filled dots indicate mean number of shifts across placements, error bars show standard deviation

– The BEAST ancestral biome reconstructions indicate that the rainforest biome is the most likely ancestral biome to all Cercidoideae (Fig. 3; probability of rainforest: 0.47). The genus *Cercis*, which is concentrated in the temperate biome, is associated with a shift from rainforest to temperate. The analyses suggest a minimum of 25 additional biome shifts (Figs. 3, 6), when we consider the most probable states reconstructed at nodes. Biome shifting from rainforest to succulent occurs five times and this type of shift occurs throughout the phylogeny (two occur in the genus *Bauhinia*, one is characteristic of *Brenierea insignis,* one subtends the genus *Adenolobus*, and one subtends the genus *Tylosema*). Five shifts in the opposite direction, from the succulent to rainforest biomes, are also observed, all within *Bauhinia*. Five shifts from rainforest to savanna can be identified: one is characteristic of the genus *Lysiphyllum*, one subtends *Piliostigma*, one occurs in a *Phanera* species, and two in *Bauhinia*. There were no shifts from the savanna biome and no shifts from the temperate biome. The BEAST analyses also suggest seven shifts from the succulent biome to mixed rainforest + savanna distributed species and three shifts from the rainforest to mixed savanna + succulent biome distributed species (all in the genus *Bauhinia*). Overall, 20 shifts occur in the Bauhinia clade and four in the Phanera clade. Biome shifts occurred between 48.74 Ma (95% HPD: 46.12—54.74 Ma) and 4.23 Ma (95% HPD: 1.88–6.93 Ma).

The ARD model was recovered as the best-fitted model for both biomes and habit based on the Akaike information criterion and was therefore used for the make.simmap and fit.discrete analyses of character evolution and phylogenetic signal.

Using stochastic character mapping as implemented in the make.simmap function (Phytools), the temperate biome is reconstructed as the most likely ancestral biome to all Cercidoideae (Fig. 4; probability temperate biome: 1.0). The common ancestor to the Cercidoideae clade, excluding *Cercis* and *Adenolobus*, is reconstructed as most likely belonging to the succulent biome. The make.simmap analyses reconstruct 21 biome shifts, the most common being from succulent to rainforest (Figs. 4, 6). A shift to the rainforest biome likely occurred in the Phanera clade, followed by more recent shifts to the savanna biome. Four types of shifts are recovered: from succulent to rainforest (10 shifts), rainforest to savanna (4 shifts), temperate to succulent (2 shifts) and succulent to savanna (2 shifts). In addition, there are three shifts out of the succulent biome to mixed rainforest + savanna distributed species (Fig. 4).

BEAST analyses estimate that the tree/shrub habit is the ancestral habit for Cercidoideae (Fig. 3; probability tree/shrub habit: 0.98), with a shift to lianescent habit in the Phanera clade (32.36 Ma; 95% HPD: 25.85—38.79 Ma), followed by five shifts back to the tree/shrub habit: in *Barklya syringifolia* F.Muell. (16.44 Ma; 95% HPD: 12.27—21.31 Ma), in *Lysiphyllum* (16.60 Ma; 95% HPD: 12.65—20.97 Ma), and in three *Phanera* species (from c. 2 Ma to 10 Ma). Within the Phanera clade, a shift from the lianescent habit to a herbaceous perennial habit occurs in *Tylosema* (22.82 Ma; 95% HPD: 16.72—28.90 Ma). In the Bauhinia clade, species are all trees or shrubs, and no shifts in habit were observed.

Using stochastic character mapping as implemented in the make.simmap function (Phytools), two types of habit shifts occurred: from liana to tree/shrub and liana to herbaceous perennial (Fig. 4). Other types of shifts were not observed in our analysis. In contrast to the BEAST reconstructions, this analysis indicates that the lianescent habit is the ancestral habit for Cercidoideae (Fig. 3; probability lianescent habit: 0.56), with shifts to the tree/shrub habit subtending *Cercis*, *Adenolobus*, *Griffonia*, *Gigasiphon*, and the Bauhinia clade, as well as the same five shifts to tree/shrub in the Phanera clade as reconstructed by BEAST (Fig. 3).

While the tree/shrub habit is found in Cercidoideae species across all four biomes, all 25 lianescent species were assigned to the rainforest biome (10 of which were attributed to both rainforest and savanna biomes; three to rainforest and succulent biomes). Of the mostly lianescent genera in the Phanera clade, two of the five shifts to the tree/shrub habit are associated with a shift in biome from rainforest to savanna, one of which implies the genus *Lysiphyllum*. In *Tylosema* the shift in growth form from lianescent to herbaceous perennial is associated with a shift from the rainforest to the succulent biome in the BEAST reconstruction (Fig. 3), but not in the make.simmap analyses where the succulent biome is the ancestral condition (Fig. 4).

### Biogeographical reconstructions

– The DEC + j model was recovered as the best-fitting model in the BioGeoBEARS analysis, with an Akaike information criterion (AIC) weight (corrected for sample size) of 0.90. Ancestral area reconstructions are therefore reported based on this model (Fig. 5).

The ancestral area reconstruction of the crown node of Cercidoideae is ambiguous. A widespread ancestor occurring in Africa, North America, and Northern Asia is the most likely reconstruction (probability 0.36), whereas a distribution in only Africa and North America or Africa and Northern Asia is also likely (both have a probability of 0.28). Reconstructions to a single area are much less probable (Africa: 0.02, North America: 0.01, Northern Asia: 0.01). However, a widespread ancestor occurring in all these regions, but not elsewhere, appears implausible given the continental configuration c. 50 Ma, so the actual distribution of the most recent common ancestor of Cercidoideae remains unclear. This uncertainty is exacerbated by the lack of a European *Cercis* species in our phylogeny, which likely has affected the reconstruction at the crown. Nevertheless, the crown and most of the oldest nodes of the sister clade of *Cercis*, which contains the vast majority of all Cercidoideae, are very strongly reconstructed as African, suggesting that much of the early evolution of the subfamily occurred in Africa (Fig. 5).

The biogeographical analysis indicates that many long-distance dispersal events have occurred throughout the evolution of lineages (Fig. 5). We recover a total of 13 cross-continent dispersal events, disregarding dispersals from North America to Central America (Fig. 5): three occur within the succulent biome (in *Bauhinia*), five within the rainforest biome (throughout the phylogeny), one occurs within the savanna biome (in *Lysiphyllum*), one is associated with the temperate biome (in *Cercis*), one is associated with a shift from the rainforest to succulent biome (in *Brenierea*), one with a switch from the rainforest to temperate biome (subtending *Cercis*), and one with a switch from the succulent to rainforest-savanna biome (in *Bauhinia viridescens*). Similarly, the biome and continent reconstruction analyses show that the 26 biome shifts are rarely associated with continental disjunctions. Lineages that disperse to other continents tend to remain within the same biome and biome shifts tend to occur within the same continents. There are two notable occurrences of a shift in both biome and continent. One is with *Brenierea* where the long distance dispersal from Africa to Madagascar in the ancestor of the genus is accompanied by a shift from the rainforest to succulent biome in the BEAST reconstruction (Fig. 5). In the make.simmap analysis because the ancestral state is the succulent biome, there is no shift in biome in *Brenierea* (Fig. 4). The other subtends the genus *Cercis* and indicates a change from a rainforest, possibly African, ancestor to a temperate North American or Northern Asian crown node. Again, this biome shift is not inferred by make.simmap, as the ancestral state is reconstructed as temperate. Furthermore, in the genus *Lysiphyllum*, there is a shift characteristic of the entire genus from a rainforest liana to a savanna tree or shrub at approximately 16.6 Ma that occurred within Asia, but that is shortly followed by a dispersal event to Australia (characteristic of the entire genus excluding *Lysiphyllum winitii* (Craib) de Wit, the sister species to the genus). Our analyses reveal only one occurrence of a shift in habit coinciding with dispersal to a new continent: the divergence of *Barklya syringifolia* (nested within the rainforest genus *Schnella*) is associated with a shift from liana to tree/shrub habit and with a dispersal from South America to Australia. The biogeographical analyses also reveal that shifts from succulent to rainforest biomes mostly occur in the New World, whereas shifts in the opposite direction occur mainly in Africa. Shifts from rainforest to savanna biomes only occur in Africa and Asia.

### Phylogenetic biome conservatism

– The numbers of biome shifts obtained with our data were significantly lower than those obtained with reshuffled data (Supplementary Information Table S1), meaning that we can reject the null hypothesis that there are as many changes in both biome and habit as we could expect if they were randomly distributed. The median Pagel’s lambda was 0.874 for biomes and 0.998 for habit (Supplementary Information Table S2). The Delta statistic was higher for our data compared to reshuffled data, for both biomes and habit (p-value < 0.05) (Table S2). These values suggest biome conservatism and habit conservatism.

## Discussion

Subfamily Cercidoideae is characterized by a primarily pantropical distribution with complex patterns across wet and dry tropical biomes. Although our analyses indicate that biomes are phylogenetically conserved in Cercidoideae, the diverse distribution patterns observed suggest both that long distance dispersal is prominent in the evolutionary history of the group and that over time Cercidoideae have been able to adapt to different environments. Comparison of the differing transcontinental distribution patterns and habits between major clades in Cercidoideae can help provide insights for understanding what determines the distribution of these clades, including the importance of factors such as dispersal limitation and the ability to adapt to new environments. To answer these questions, we looked at the prevalence of biome shifts throughout the history of Cercidoideae and the relative frequencies of shifts between different biome types (Fig. 6). Our analyses show that biome shifts have occurred throughout the evolution of the subfamily and on almost all continents where Cercidoideae species are found. However, these distribution patterns differ between the two major tropical clades, the Phanera and Bauhinia clades, and the sister lineages to these two clades. Below we discuss the observed patterns in each of these lineages, with an attempt to dissect out the relative importance of biome switching, oceanic dispersal and growth form in the evolution of Cercidoideae.

### Growth form evolution and biome shifts

– Phanera clade species are found in Africa, Asia, Australia, South America and Central America and are mostly rainforest lianas (approximately 75%), except for *Tylosema,* a succulent biome herbaceous perennial genus, and *Lysiphyllum,* a savanna genus of mostly tree and shrub species, but that sometimes are semi-scandent and climbing, with or without tendrils (Wunderlin et al. 1987; Lewis and Forest 2005). Bauhinia clade species are similar to the Phanera clade in their widespread geographic distribution. Even though they also are found in all three major tropical biomes, the number of Bauhinia clade species in each of these biomes is more evenly distributed and phylogenetically less structured than that observed in the Phanera clade. In addition, Bauhinia clade species are more uniform in habit, being all small trees or shrubs, without tendrils (Wunderlin et al. 1987). Both clades are biogeographically structured, with most of the American, the Asian and the African species grouping into clades, as previously noted (Meng et al. 2014; Sinou et al. 2020), suggesting a similar dispersal limitation in these two major groups even though long-distance oceanic dispersal has occurred repeatedly over the history of the subfamily. However, the arborescent Bauhinia clade appears to have more readily shifted between wet and dry habitats (20 or 14 times, Figs. 3–4), in comparison to the mostly rainforest lianas typical of the Phanera clade (three or four biome shifts, Figs. 3–4) refuting our initial hypothesis that lianescent species would have shifted biomes more frequently than non-lianescent lineages.

Donoghue and Edwards (2014) proposed that the presence of certain “enabler” traits could be a significant factor influencing the probability of niche evolution and Gagnon et al. (2019) noted that evolutionary shifts in key functional traits, such as growth form, deciduousness and tolerance to frost, drought and fire, are often necessary to overcome adaptive barriers delimiting biomes. For example, Lohmann et al. (2013) suggested that the variety of both wet and dry niches occupied by a neotropical clade of Bignoniaceae species that occur in the savanna, succulent and rainforest biomes, could be related to variation in growth forms in the lineage studied, which includes both shrubby and lianescent species. Lohmann et al. (2013) also remarked that this type of variation in habit is otherwise relatively rare in neotropical plant groups. This contrasts with our observation that the Bauhinia clade, which is more uniform in habit, is found in as many different biomes as the more variable Phanera clade, but has switched biomes much more frequently than the Phanera clade, suggesting that growth form may not be the driving factor or enabler behind wide biome distribution in Cercidoideae.

### Tropical biome shifts

– In addition to these overall patterns of biome distribution in the two tropical clades, the analyses suggest that only four types of biome shifts have occurred in their evolutionary history. Shifts from the rainforest to succulent biomes (only observed in the BEAST analyses; Fig. 3) occur from the early Eocene to late Miocene, and all occur within tree/shrub lineages of Africa, except for one shift within the genus *Bauhinia* that occurs within South America and one shift in the African genus *Tylosema,* which coincides with a change to a dry-adapted herbaceous perennial habit (some species with tendrils). One of the significant shifts observed is within the Bauhinia clade in Africa (at c. 20 Ma), which is followed by a subsequent long-distance dispersal to South America, where the genus appears to have diversified leading to primarily succulent biome lineages in Central America and Mexico. Some of these shifts out of the rainforest biome may have been driven by the climate cooling that occurred during the Oligocene and changed the global distribution of biomes, including retraction of rainforests, resulting in less connectivity between Old World and New World tropical forests (Morley 2011; Landis et al. 2020). The onset of the Paleocene Eocene Thermal Maximum to the end of the Miocene was an important geological period for diversification of plants, including Leguminosae, due to the global cooling and increased seasonality (Jaramillo et al. 2010; Hughes et al. 2015). The rainforest Phanera clade appears to show connectivity between the African ancestral lineages and South America (*Schnella*) and Asia (*Phanera*-*Cheniella*) with dispersals in the Miocene, suggesting again that this clade is strongly adapted and more constrained to the rainforest biome. However, in the Bauhinia clade, the ancestral African-Asian rainforest lineages have switched to dryer biomes in Africa and remained in the dryer biomes after dispersal to South America, at about the same time. Interestingly this contrasting pattern suggests the rainforest biome species of the Phanera clade may be more biome conserved and that, as suggested by Donoghue (2008; see also Edwards and Donoghue (2013); Donoghue and Edwards (2014); Pyron et al. (2015)), and as found in other wet tropical plant clades (Richardson et al. 2001; Roncal et al. 2013; Eiserhardt et al. 2013; Weeks et al. 2014), it would be easier to move than to adapt to changing climatic conditions. Similarly, Ringelberg et al. (2023) reported that for Mimoseae, climatic distance is more important than geographic distance in explaining species distributions within all continents (except Asia where the climate is more uniform). This contrasts with the Bauhinia clade, in which biome switching more often has occurred within the same continent indicating that it would seem to have been easier to change from wet to dry forest environments than to disperse to new continents.

Shifts in the opposite direction, from the succulent to rainforest biomes, occur almost only in the New World and are the most recent types of shifts in Cercidoideae (middle Miocene-Pliocene). Our results (Fig. 6), which indicate a total of 12 or 15 shifts (Figs. 3–4) out of the succulent biome, contrast with several studies that have provided evidence for succulent biome conservatism (Lavin et al. 2004; Pennington et al. 2009; Gagnon et al. 2019; Ringelberg et al. 2020), noting that plant lineages adapted to this biome may be ecologically constrained and have a tendency to remain in the succulent biome over time (and across continents), although shifts from succulent to wet biomes have been documented in other lineages (e.g., Boluda et al. 2022). Almost all the shifts observed occur in the arborescent *Bauhinia* genus, again suggesting a possible ecological lability in this clade that may otherwise be unusual in tropical plants. However, we observe that the African-New World clade in the Bauhinia clade is indeed mostly confined to the succulent biome, with incursions back into (or possibly in situ adaptation to) the rainforest biome for individual species or species pairs in South and Central America. The Bauhinia clade follows the pattern observed in other studies of the succulent biome, with a Miocene radiation linked to Oligocene and Miocene global cooling and expansion of drought-prone vegetation (Arakaki et al. 2011; Gagnon et al. 2019; Ringelberg et al. 2020). This transcontinental succulent biome clade with mid to late-Miocene age dispersals may thus correspond to the phylogenetic pattern observed in other succulent biome transcontinental legume clades (Lavin et al. 2004; Gagnon et al. 2019) despite a number of shifts into wetter habitats.

Shifts from rainforest to savanna biomes occur in both Asia and Africa (with one possible shift reconstructed in South America, Fig. 4), at varying times and in both major tropical clades. Many other studies have indicated frequent and recent shifts in plant lineages between moist forests and savanna (Schrire et al. 2005; Simon et al. 2009; Estrella et al. 2017; Antonelli et al. 2018; Gagnon et al. 2019; Gorel et al. 2022). Shifts between these two biome types were shown to be some of the most common types of shifts in Detarioideae (Estrella et al. 2017; similar age to Cercidoideae) and in the Pterocarpus clade (Schley et al. 2022), which also occur in Africa and Asia. The expansion of grasslands during the late Miocene and Pliocene has been suggested as a driver of these shifts from wet biomes to savannas (Donoghue and Edwards 2014) and could also help explain shifts of this type in Cercidoideae. Two of five of these shifts involve changes in habit from liana to tree/shrub. Our BEAST analysis (Fig. 3) shows one early occurring shift (early Oligocene) from a tree/shrub habit to a lianescent habit in the Phanera clade, followed by many more recent shifts back to the tree/shrub habit (in *Lysiphyllum*, *Barklya* and some *Phanera* species) or a change to the dry-adapted herbaceous perennial habit (in *Tylosema*). This could suggest that it is easier to shift from a liana to another habit but difficult for tree/shrub lineages to shift habits (and this could be consistent with the observed stability in habit throughout the evolution of the Bauhinia clade lineages which remain trees or shrubs). Furthermore, in *Lysiphyllum* which generally are considered trees or shrubs, several species have been reported as climbing (Wunderlin 2010), suggesting that some species retain the ancestral ability to climb under particular environmental conditions, and as noted by Rowe and Speck (2005) plasticity in growth form is not unexpected. The shift back to the tree/shrub habit in *Lysiphyllum* (and in *Phanera retusa*) could suggest that the lianescent habit is not well adapted to the fire-prone savanna biome and that shifts to the savanna biome are more likely for species with a tree habit. However, in the case of *Lysiphyllum* and *Tylosema,* for example, our analyses do not allow us to conclude whether biome shifts occur before habit shifts, or vice versa. In addition, our results contrast with those of Gagnon et al. (2019) who showed that a shift to the lianescent habit from the ancestral tree/shrub habit is mostly coincidental with a shift from the succulent to savanna biome in the Caesalpinieae. The association between growth form and biome is also nuanced by Gorel et al. (2022), who studied biome shifts at the generic level in multiple plant lineages in Africa, and observed that even though lianas tend to occur in forest environments whereas tree and shrubby species are common in savannas, in many clades the ancestral habit is retained even after shifts into the savanna biome.

Finally, although succulent to savanna biome shifts are observed (Figs. 3–4), almost all these shifts subtend widespread species occurring in both rainforest and savanna biomes (counted as half shifts to either biome in the upper part of Fig. 6). These succulent to savanna biome shifts are only found in the Bauhinia clade, and in all but a single case involve recently diverged species. As discussed above, these results, as well as the complete absence of shifts from savanna to any other biome (Fig. 6), chime with the recent expansion of grasslands across the tropics (Donoghue and Edwards 2014). They also support previous findings that adaptations to fire are relatively straightforward to evolve in tropical trees (Simon et al. 2009; Simon and Pennington 2012), although the more frequent shifts from wet forests than from dry forests to savannas (Fig. 6) suggest that not all lineages are equally likely to evolve such fire adaptations. While the make.simmap analysis (Fig. 4) reconstructs shifts from succulent biome to savanna as occurring in multiple continents, the BEAST analysis (Fig. 3) suggests that all but one of these shifts happened in South America, likely reflecting the recent assembly of the Cerrado, the large, fire-prone savanna in central Brazil (Simon et al. 2009).

### Drivers of biome shifts

– Spatial adjacency or connectivity of biomes has been suggested as a factor that could increase the probability of shifts between biomes, due in part to the higher geographic opportunity for shifts and because adjacent biomes are often more climatically similar (Donoghue and Edwards 2014). However, even though adjacent in many parts of the Americas, the succulent and rainforest biomes are climatically distinct, and yet we observe shifts in both directions between the two biomes. Conversely, although the succulent and savanna biomes are adjacent and share similarities in climate, both being seasonally dry (Pennington et al. 2009; Särkinen et al. 2011; Ringelberg et al. 2020), only a few shifts between these two biomes are observed in Cercidoideae, all from succulent to savanna (Fig. 6). This suggests that spatial adjacency is not a major factor driving or preventing biome shifts in Cercidoideae. Instead, and as noted in other studies, shifts from succulent to savanna biomes could be limited by a lack of adaptations in succulent biome species to the frequent fires that are characteristic of the savanna biome, such as thick corky bark and the ability to resprout from rhizomes (Pennington et al. 2009; Simon et al. 2009; Simon and Pennington 2012). Interestingly, *Tylosema* species, which are dry-adapted herbaceous perennials with a fleshy caudex that dies back and occur in the succulent biome or a mix of succulent and savanna biomes in Africa, may indeed be better adapted to a fire-prone biome.

Another factor that has been suggested as having an influence on the frequency of biome shifts is the age of biomes and their connectivity through time (Donoghue and Edwards 2014). The rainforest biome is suggested to have originated in the Cretaceous-Eocene (Morley 2011; Landis et al. 2020) and the succulent biome in the Oligocene (Gagnon et al. 2019), whereas the savanna biome appeared in the late Miocene-early Pliocene (Simon et al. 2009; Edwards et al. 2010; Pennington and Hughes 2014). In Cercidoideae there have been relatively few switches to the savanna biome, either from the rainforest or succulent biome (Fig. 6). Other than in the Australian *Lysiphyllum* where the genus seems to have diversified after dispersal from Asia at about 10 Ma, the other shifts concern recent single or few species lineages, at least with the current phylogenetic sampling (*Piliostigma* also appears to be a mostly savanna-adapted genus of Africa and Asia, but some species occur in both the rainforest and savanna biomes and only two species are included in our phylogeny). Furthermore, there is a complete absence in either of our analyses of switches from the savanna to any of the other biomes (Fig. 6). Our results therefore strongly support the age of biomes as an important factor in determining the direction of biome shifts.

### Long distance dispersal and phylogenetic biome conservatism

– Overall, for the two main tropical clades, our ancestral character reconstruction analyses show that biome shifts tend to occur within the same continent and that dispersals to new continents tend to occur within the same biome. This appears to be true across different continents and biomes, except in the case of the genus *Lysiphyllum* in which the shift from the rainforest to the savanna biome that subtends the entire genus is followed by dispersal from Asia (*L. wintii* (Craib) de Wit) to Australia (all other species). This biome shift in *Lysiphyllum* also coincides with a shift from a lianescent to a semi-scandent shrubby habit (with some species having tendrils). One other transcontinental shift associated with a change in biome is observed in the monospecific Madagascan succulent biome genus *Brenierea*, which derives from an African rainforest ancestor in the BEAST reconstruction, but from a succulent biome ancestor in the make.simmap analysis (Figs. 3, 4). Our analyses indicate four inter-continental dispersals within the succulent biome, five within the rainforest biome and one within the savanna biome, highlighting the tendency expressed by Donoghue (2008) that it may be easier for plants to move than to adapt, even across large distances. The multiple long-distance dispersal events between continents reconstructed in Cercidoideae, as well as in other legume lineages such as the Pterocarpus clade (Schley et al. 2022), Detarioideae (Estrella et al. 2017), and Mimoseae (Ringelberg et al. 2023), emphasize the importance of trans-oceanic dispersal events in shaping distribution patterns of pantropical clades. Nevertheless, oceans pose a formidable barrier to dispersal, and are likely a major factor shaping the distribution of these lineages at a pantropical scale (Ringelberg et al. 2023).

In Cercidoideae, lineages may more easily adapt to environmental changes than in some other tropical clades and floras, as reflected in a slightly higher proportion of biome switches per speciation event (mean 11–15%; Fig. S2) than observed in Mimoseae (5% per speciation event (Ringelberg et al. 2023)) and in other plant lineages (e.g., 7% per speciation event in African lineages (Gorel et al. 2022); 4% in Southern Hemisphere lineages (Crisp et al. 2009)). This may reflect an overall lower level of phylogenetic biome conservatism in Cercidoideae, but the absence of large radiations within the same biome in this subfamily likely also plays a role. Several such radiations have occurred in Mimoseae, for example resulting in c. 300 *Inga* species predominantly in Neotropical rainforests, and c. 1000 *Acacia* species in the dry regions of Australia (Ringelberg et al. 2023). However, the trajectory of biome switches through time can be assessed in different ways, and if the number of switches per million years is used as a metric, Cercidoideae and Mimoseae are largely indistinguishable (Fig. S2). Comparisons between Cercidoideae and Mimoseae are further complicated because the results in Mimoseae are based on switches between precipitation niches rather than biomes (Ringelberg et al. 2023). Detailed, standardised comparisons of biome switches through time in Cercidoideae, Mimoseae, and other pantropical species-rich and ecologically diverse plant lineages are required to more to fully tease apart the roles of phylogenetic biome conservatism and evolution in shaping richness and distribution patterns in different lineages.

### Relictual temperate and species-depauperate lineages

– As in other studies examining ancestral biome reconstructions (e.g., Spriggs et al. 2015; Landis et al. 2021), our results are equivocal as to the identity of the ancestral node. The distribution of *Cercis*, which is sister to the rest of the subfamily, spans dry to mesic climates in the Northern Hemisphere (Davis et al. 2002; Fritsch et al. 2018) but is reconstructed as having originated in the rainforest biome (BEAST analysis, Fig. 3). This would suggest that at least one shift has occurred early-on in the evolution of Cercidoideae between rainforest and temperate biomes, possibly through an intermediary ancestor that is no longer extant (considering that our time-calibrated phylogeny shows that c. 45 Ma separate the basal and crown nodes of *Cercis*). However, the results are not conclusive and stochastic character mapping analyses (Fig. 4) reconstructed the temperate biome as ancestral for Cercidoideae and *Cercis*. This uncertainty about the ancestral biome state at the root of the tree, as well as the significant number of other differences between the BEAST and make.simmap reconstructions (Fig. 6), reflect the difficulties of attempting to reconstruct events that happened in the deep evolutionary past, especially using only information from extant species (Marshall 2017).

Cercidoideae fossils, which span the Eocene to Pliocene, have been found in multiple localities in both the New World and Old World, with *Cercis* fossils concentrated at northern latitudes, and *Bauhinia* s.l. fossils occurring mostly within 20 degrees latitude of the equator (Fig. 6). However, Oligocene (and one Eocene) fossils of both *Bauhinia* s.l. and *Cercis* in southern China may suggest a more widespread distribution of *Cercis* at that time when climatic conditions at northern latitudes were much warmer than currently. Tropical rainforests and temperate forests were more interconnected during the Eocene than in more recent eras (Morley 2011), which could help explain this shift in Cercidoideae during this time as well as the absence of shifts between these two biomes in subsequent diversifications in Cercidoideae. Our analyses also suggest that contrary to *Bauhinia* s.l., *Cercis* species would have better adapted to cooling climatic conditions explaining their continued presence at temperate latitudes. Fritsch et al. (2018) suggested that *Cercis* could have avoided extinction (unlike other taxa in the subfamily) through leaf adaptations to xeric and mesic habitats. A similar pattern of early branching woody temperate lineages sister to diverse tropical clades as observed in Cercidoideae is seen in subfamily Caesalpinioideae (Schnabel et al. 2003; Bruneau et al. 2008; Ringelberg et al. 2022). This contrasts with the vast majority of temperate legume species which are often herbaceous (or functionally so) and derived from tropical lineages (e.g., Wojciechowski et al. 2000, 2004; Cardoso et al. 2013; Oyebanji et al. 2023). These old temperate woody lineages of Cercidoideae and Caesalpinioideae are also notably species-poor and represent classic examples of depauperons (Donoghue and Sanderson 2015), as might be expected for relics. These may represent relictual lineages from previous richer boreotropical floras that occupied temperate regions during the Eocene when tropical lineages dominated these areas, supported in part by the presence of fossils of Cercidoideae dating to the Eocene at higher latitudes (Fig. 1D) and by a long branch between the stem and crown node of *Cercis*. Lineages such as *Cercis* and *Adenolobus*, as well as *Gleditsia*, *Gymnocladus* and *Umtiza* (Caesalpinioideae) may have managed to adapt to the cold as the tropics contracted from the Oligocene onwards perhaps suffering extinction along the way and hence accounting for how species-poor they are. This scenario parallels the ‘dying embers’ hypothesis formulated by Spriggs et al. (2015) to account for ancestral lineages in which speciation rates have decreased and extinction rates have increased towards the present.

The genus *Adenolobus,* which is restricted to the succulent biome of south-western Africa, is sister to the remainder of Cercidoideae, except *Cercis*, and represents another early diverging lineage of small trees or shrubs that is somewhat more geographically isolated from the rest of Cercidoideae. *Adenolobus* is also species-poor and may represent a vestigial lineage that has poorly adapted to changing environmental conditions, including climate. Schrire et al. (2005) suggested that legumes originated in a dry tropical biome (succulent biome) around the Tethys Sea in the early tertiary and that lineages subsequently shifted to moist tropical forests. Although this pattern does not seem to be supported by our analyses, the presence of an early diverging succulent biome lineage in Cercidoideae further complicates ancestral biome reconstructions and adds caution to the conclusion that Cercidoideae is of wet tropical origin. The pattern seen here is also similar to that seen in Caesalpinioideae, where the succulent biome South African endemic *Umtiza* is phylogenetically sister to the mostly north temperate genera *Gleditsia* and *Gymnocladus* (Manzanilla and Bruneau 2012; Ringelberg et al. 2022).

In summary our analyses suggest that several biome shifts have occurred throughout the evolution of Cercidoideae lineages between the rainforest and succulent biomes, from the rainforest and succulent to the savanna biome, and also possibly from the rainforest to the temperate biome. Shifts between the rainforest and succulent biomes have likely been the most common, whereas shifts to the relatively young savanna biome are rarer, suggesting a more important role for biome age than spatial proximity and connectivity in shaping the direction and prevalence of biome shifts. While our analyses show two major concomitant shifts in biome and habit (*Lysiphyllum* and *Tylosema*), we do not find that lianescent lineages have shifted biomes more frequently. Indeed, biome shifts have occurred between all three tropical biomes in the non-lianescent Bauhinia clade, and (based on our species sampling) five times more frequently than in the habit-plastic Phanera clade, which contains lianas, tree/shrubs, and herbaceous species. We also find that while biome shifts tend to occur within continents, several long-distance dispersals to new continents have occurred, most often within the same biome. The frequent shifts in Cercidoideae indicate that species have been able to adapt to significantly different environments over time, but that although *Cercis* appears to have adapted to the frost-prone temperate environment, other Cercidoideae genera clearly have remained in tropical biomes even though some species occur geographically close to temperate zones. The question remains whether species capable of shifting biomes have adapted in situ, or whether shifts to new biomes were facilitated by pre-existing adaptations (i.e., exaptations) such as more suitable habits for the new environment. Cercidoideae may provide a classic example of a tropical arborescent group, which overall was poorly adapted to colder climates and flourished in tropical zones with relictual temperate and species-depauperate lineages occurring sister to the tropical clades. The contrasting patterns observed in the two tropical sister clades of similar Oligocene age (i.e., the Phanera and Bauhinia clades) also provides an ideal model for further study of which biotic and abiotic factors are important in determining the prevalence of biome shifts through time.

## Supplementary Information

Below is the link to the electronic supplementary material.

Supplementary file1 (DOCX 903 kb)

## Data Availability

The datasets generated and/or analysed during the current study are available in the Zenodo repositories; Aligned concatenated DNA sequence matrix for four loci (10.5281/zenodo.13256230), BEAST MCC phylogenetic tree (10.5281/zenodo.13227927), BEAST input data file for biomes and habits (10.5281/zenodo.13227921), Excel spreadsheet with biome, habit and continent assignment for 107 Cercidoideae species (10.5281/zenodo.13227904) and occurrence data sets for all Cercidoideae species analysed (10.5281/zenodo.13256199).
