# Supplementary material for: Biome evolution in subfamily Cercidoideae (Leguminosae): a tropical arborescent clade with a relictual depauperate temperate lineage
Source: Rev Bras Bot. 2024 Dec 18;48(1):11. doi: 10.1007/s40415-024-01058-z (PMC11652589; doi:10.1007/s40415-024-01058-z)

**Supplemental Information**

**Supplemental Table S1.** Number of biome and habit shifts in Cercidoideae obtained with real and reshuffled data using stochastic character mapping as implemented in phytools (make.simmap function) and an all rates different (ARD) model (best-fitted model for biomes and habit). Numbers of shifts using real data were obtained using a sample of 100 trees and 100 make.simmap simulations. Numbers of shifts with reshuffled data were obtained by reshuffling the data five times per tree, using 10 trees and 10 make.simmap simulations. 1st quartiles, 3rd quartiles and medians of numbers of shifts are shown in this Table.

|  | Biomes | | Habit | |
| --- | --- | --- | --- | --- |
|  | Real data | Reshuffled data | Real data | Reshuffled data |
| 1st quartile | 29.175 | 506.6 | 11.335 | 565.075 |
| Median | 30.155 | 964.9 | 11.84 | 1146.25 |
| 3rd quartile | 32.47 | 1041.4 | 13.2375 | 54665.025 |

**Supplemental Table S2.** Phylogenetic signal for biomes and habit using Pagel’s lambda (Pagel 1999) and the Delta statistic (Borges et al. 2019) calculated using an ARD model (best-fitted). Pagel’s lambda for biomes was obtained by reshuffling the biome data 100 times and calculating the median of these 100 trees. Pagel’s lambda for habit was obtained without reshuffling the data. The Delta statistics for biomes and habit were obtained using our data and using reshuffled data (100 times for both biomes and habit), then calculating a p-value as the number of times that the Delta statistic for reshuffled data is superior to the Delta statistic for observed data. A p-value of less than 0.05 indicates conservatism for a trait. P-values are null for both biomes and habit because the Delta statistics for reshuffled data were always inferior to the Delta statistics for observed data.

|  | Biomes | Habit |
| --- | --- | --- |
| Median Pagel’s lambda | 0.874 (ARD model); 0.900 (ER model) | 0.998 (ARD model) |
| Delta statistic for real data | 3.380 | 39.736 |
| Median Delta statistic for reshuffled data | 0.431 | 1.074 |
| P-value for Delta statistic | 0 | 0 |

**Supplemental Information, Figure S1**. Time-calibrated phylogeny of Cercidoideae (maximum clade credibility tree), generated in BEAST v. 2.6.3. Values at nodes represent estimated node ages with blue bars indicating 95% highest posterior density age estimates and values above branches representing Bayesian posterior probability values. Time scale is displayed below in millions of years.

**Supplemental Information, Figure S2**. Biome shifts through time in Cercidoideae (black lines) and major precipitation niche shifts through time in Mimoseae (Caesalpinioideae; red lines). Top figures show numbers of shifts relative to numbers of speciation events (nodes) per five million years, with shifts placed halfway along branches. Bottom figures show numbers of shifts relative to total amount of branch lengths per five million years, with shifts randomly placed 1,000 times along their branches. Dots are mean numbers of shifts across 1,000 placements, error bars show standard deviation. Left figures are based on BEAST reconstructions, right figures on make.simmap reconstructions. Mimoseae data are derived from Ringelberg et al. (2023).

**Supplemental information, Figs S3—S16** Distribution maps for species of Cercidoideae, for each of the 14 genera, showing occurrences on the modified biome map (Fig. 2). The savanna biome (modified from Lehmann et al. (2019); in yellow), the succulent biome (modified from Ringelberg et al. (2019); in red), the rainforest biome (Corlett and Primack (2011); in green) and regions not covered by these three biomes (in grey). Each point represents an occurrence based on an herbarium specimen.

**Supplemental information, Fig. S3.** Distribution of the two species of *Adenolobus* across biomes. The savanna biome (modified from Lehmann et al. (2019); in yellow), the succulent biome (modified from Ringelberg et al. (2019); in red), the rainforest biome (Corlett and Primack (2011); in green) and regions not covered by these three biomes (in grey). Each point represents an occurrence based on an herbarium specimen (total of 137 points).


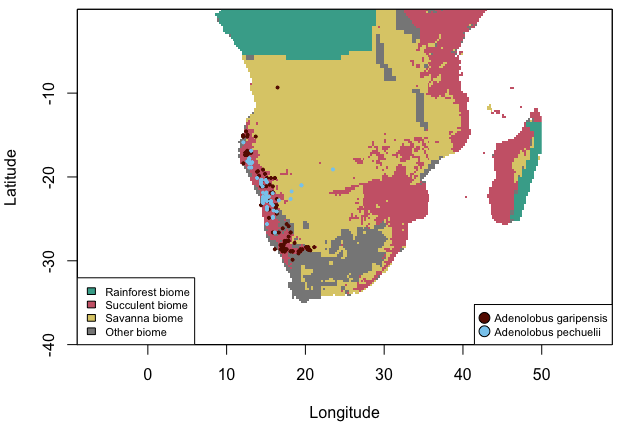


**Supplemental information, Fig. S4.** Distribution of the single species of *Barklya* across biomes. The savanna biome (modified from Lehmann et al. (2019); in yellow), the succulent biome (modified from Ringelberg et al. (2019); in red), the rainforest biome (Corlett and Primack (2011); in green) and regions not covered by these three biomes (in grey). Each point represents an occurrence based on an herbarium specimen (total of 71 points).


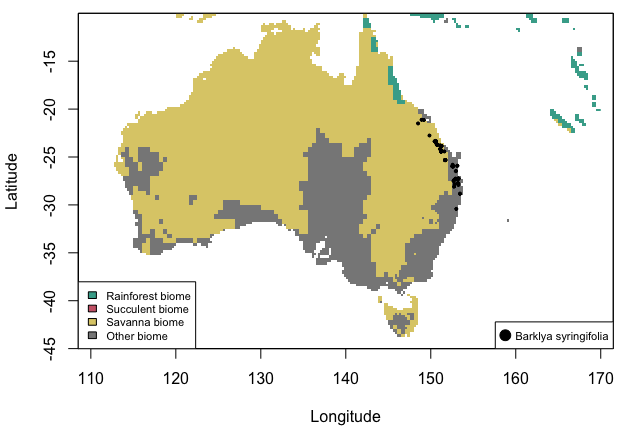


**Supplemental information, Fig. S5.** Distribution of *Bauhinia* species across biomes. The savanna biome (modified from Lehmann et al. (2019); in yellow), the succulent biome (modified from Ringelberg et al. (2019); in red), the rainforest biome (Corlett and Primack (2011); in green) and regions not covered by these three biomes (in grey). Each point represents an occurrence based on an herbarium specimen (total of 16092 points for 166 species included of a total of c. 195).


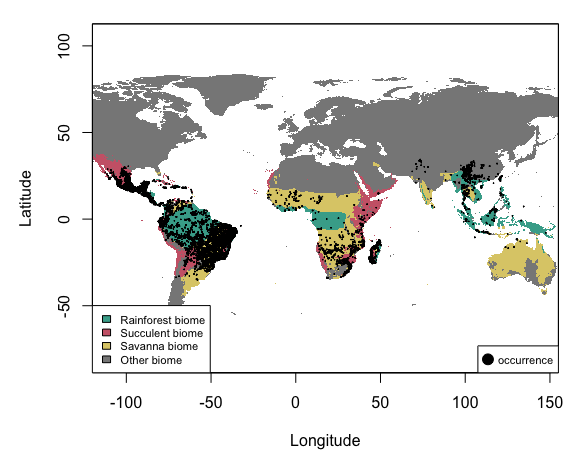


**Supplemental information, Fig. S6.** Distribution of the single species of *Brenierea* across biomes. The savanna biome (modified from Lehmann et al. (2019); in yellow), the succulent biome (modified from Ringelberg et al. (2019); in red), the rainforest biome (Corlett and Primack (2011); in green) and regions not covered by these three biomes (in grey). Each point represents an occurrence based on an herbarium specimen (total of 35 points).


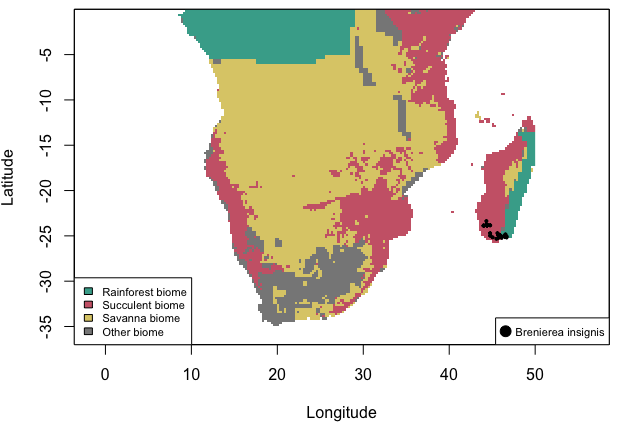


**Supplemental information, Fig. S7.** Distribution of the 10 species (and subspecies) of *Cercis* across biomes. A) New World species. B) Old World species. The savanna biome (modified from Lehmann et al. (2019); in yellow), the succulent biome (modified from Ringelberg et al. (2019); in red), the rainforest biome (Corlett and Primack (2011); in green) and regions not covered by these three biomes (in grey). Each point represents an occurrence based on an herbarium specimen (total of 3156 points).

A)

B)
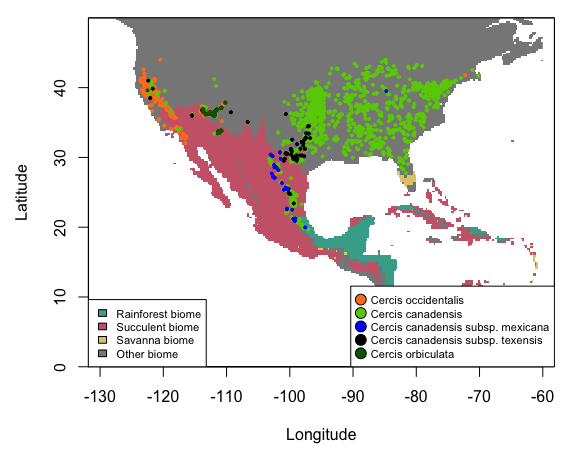

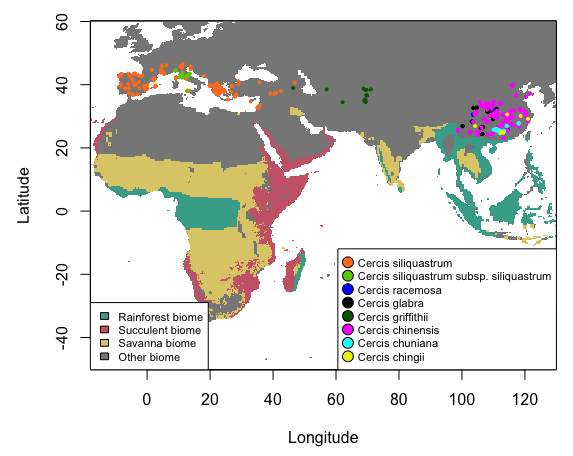


**Supplemental information, Fig. S8.** Distribution of nine species of *Cheniella* across biomes (one missing). The savanna biome (modified from Lehmann et al. (2019); in yellow), the succulent biome (modified from Ringelberg et al. (2019); in red), the rainforest biome (Corlett and Primack (2011); in green) and regions not covered by these three biomes (in grey). Each point represents an occurrence based on an herbarium specimen (total of 288 points).


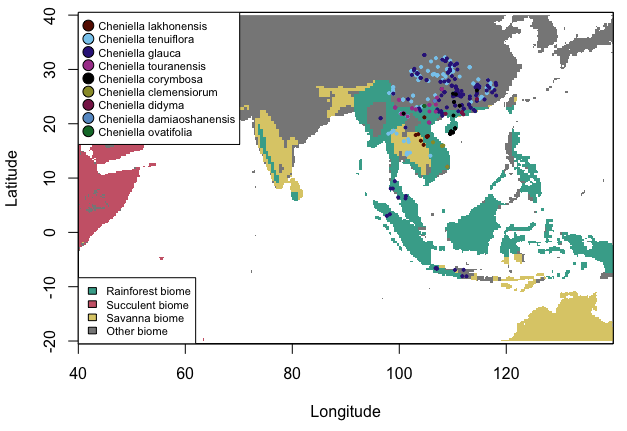


**Supplemental information, Fig. S9.** Distribution of four species of *Gigasiphon* across biomes (one missing). The savanna biome (modified from Lehmann et al. (2019); in yellow), the succulent biome (modified from Ringelberg et al. (2019); in red), the rainforest biome (Corlett and Primack (2011); in green) and regions not covered by these three biomes (in grey). Each point represents an occurrence based on an herbarium specimen (total of 40 points).


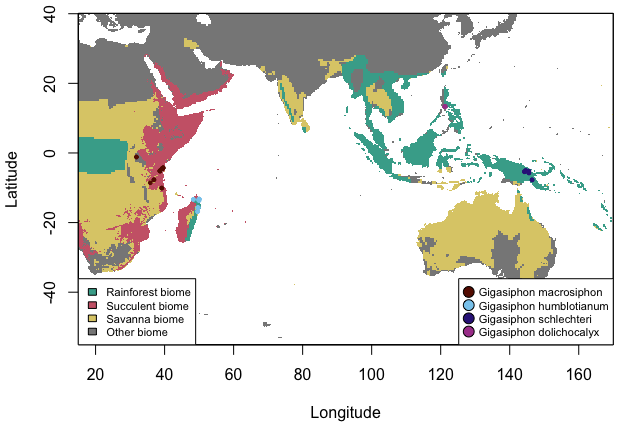


**Supplemental information, Fig. S10.** Distribution of the four species of *Griffonia* across biomes. The savanna biome (modified from Lehmann et al. (2019); in yellow), the succulent biome (modified from Ringelberg et al. (2019); in red), the rainforest biome (Corlett and Primack (2011); in green) and regions not covered by these three biomes (in grey). Each point represents an occurrence based on an herbarium specimen (total of 191 points).


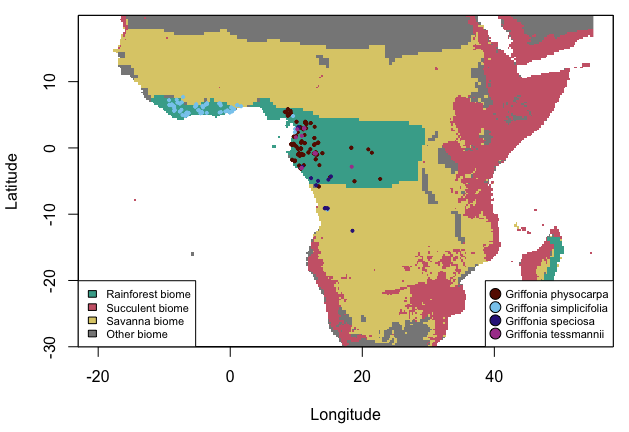


**Supplemental information, Fig. S11.** Distribution of the nine species of *Lysiphyllum* across biomes. The savanna biome (modified from Lehmann et al. (2019); in yellow), the succulent biome (modified from Ringelberg et al. (2019); in red), the rainforest biome (Corlett and Primack (2011); in green) and regions not covered by these three biomes (in grey). Each point represents an occurrence based on an herbarium specimen (total of 1107 points).


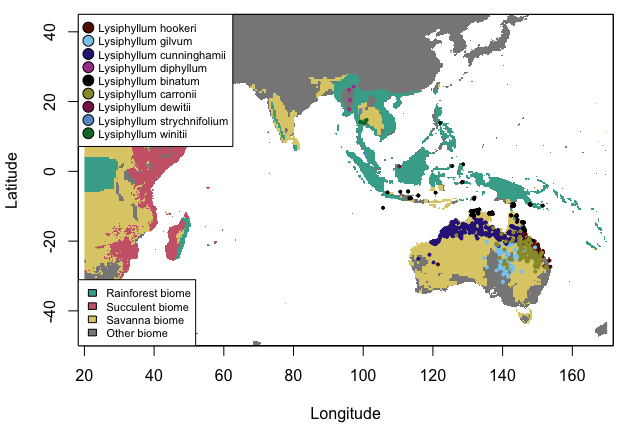


**Supplemental information, Fig. S12.** Distribution of *Phanera* species across biomes. The savanna biome (modified from Lehmann et al. (2019); in yellow), the succulent biome (modified from Ringelberg et al. (2019); in red), the rainforest biome (Corlett and Primack (2011); in green) and regions not covered by these three biomes (in grey). Each point represents an occurrence based on an herbarium specimen (total of 1460 points for 73 species included of a total of 103).


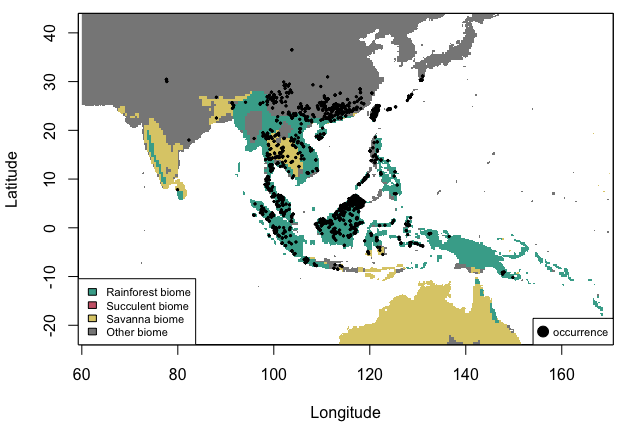


**Supplemental information, Fig. S13.** Distribution of the three species of *Piliostigma* across biomes (two missing). The savanna biome (modified from Lehmann et al. (2019); in yellow), the succulent biome (modified from Ringelberg et al. (2019); in red), the rainforest biome (Corlett and Primack (2011); in green) and regions not covered by these three biomes (in grey). Each point represents an occurrence based on an herbarium specimen (total of 366 points).


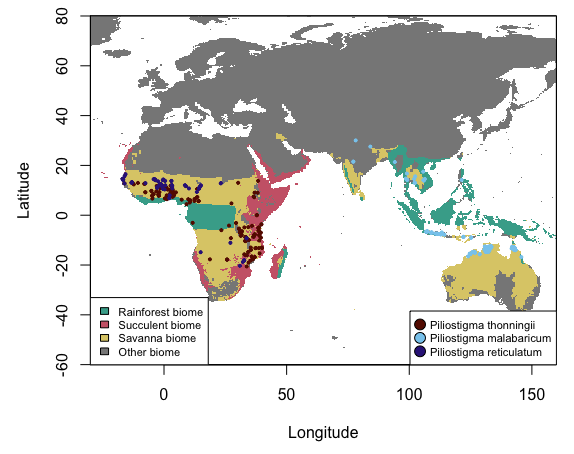


**Supplemental information, Fig. S14.** Distribution of *Schnella* species across biomes. The savanna biome (modified from Lehmann et al. (2019); in yellow), the succulent biome (modified from Ringelberg et al. (2019); in red), the rainforest biome (Corlett and Primack (2011); in green) and regions not covered by these three biomes (in grey). Each point represents an occurrence based on an herbarium specimen (total of 4142 points for 47 species).


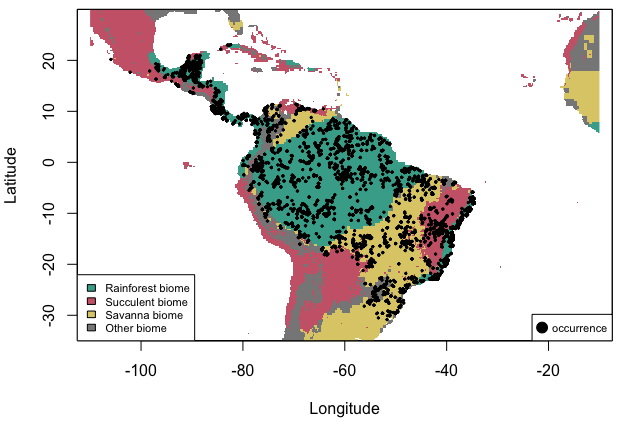


**Supplemental information, Fig. S15.** Distribution of the single species of *Tournaya* across biomes. The savanna biome (modified from Lehmann et al. (2019); in yellow), the succulent biome (modified from Ringelberg et al. (2019); in red), the rainforest biome (Corlett and Primack (2011); in green) and regions not covered by these three biomes (in grey). Each point represents an occurrence based on an herbarium specimen (total of 25 points).


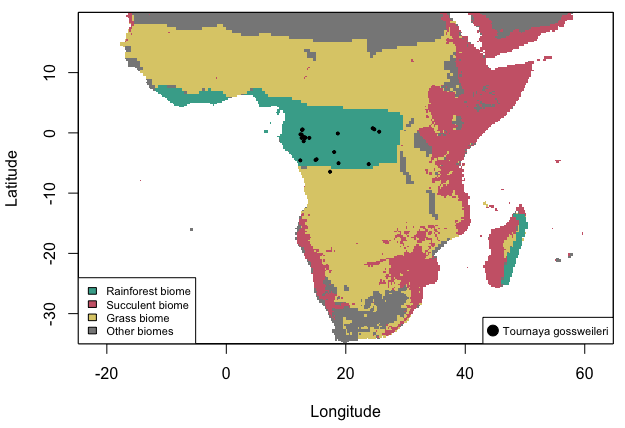


**Supplemental information, Fig. S16.** Distribution of four *Tylosema* species across biomes (one missing). The savanna biome (modified from Lehmann et al. (2019); in yellow), the succulent biome (modified from Ringelberg et al. (2019); in red), the rainforest biome (Corlett and Primack (2011); in green) and regions not covered by these three biomes (in grey). Each point represents an occurrence based on an herbarium specimen (total of 181 points).


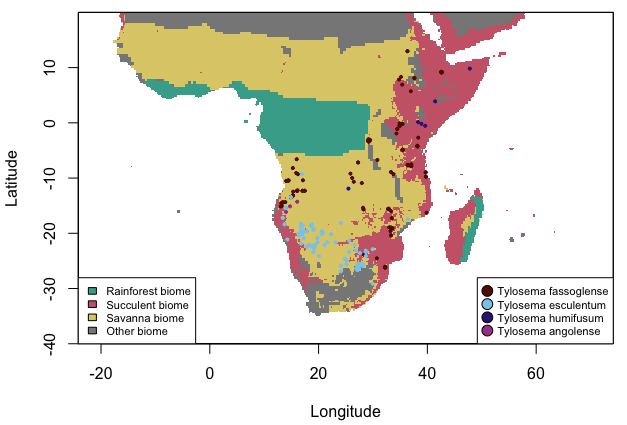

Supplement: Supplementary file 1 — Supplementary file1 (DOCX 903 kb) [file 40415_2024_1058_MOESM1_ESM.docx]
